# Landslide Susceptibility Assessment Using Integrated Deep Learning Algorithm along the China-Nepal Highway

**DOI:** 10.3390/s18124436

**Published:** 2018-12-14

**Authors:** Liming Xiao, Yonghong Zhang, Gongzhuang Peng

**Affiliations:** 1 Department of Information and Communication, Nanjing University of Information Science and Technology, Nanjing 210044, China; 20161118086@nuist.edu.cn (L.X.); zyh@nuist.edu.cn (Y.Z.); 2 Engineering Research Institute, University of Science and Technology Beijing, Beijing 100083, China

**Keywords:** landslide susceptibility, China-Nepal Highway, machine learning, LSTM, remote sensing images

## Abstract

The China-Nepal Highway is a vital land route in the Kush-Himalayan region. The occurrence of mountain hazards in this area is a matter of serious concern. Thus, it is of great importance to perform hazard assessments in a more accurate and real-time way. Based on temporal and spatial sensor data, this study tries to use data-driven algorithms to predict landslide susceptibility. Ten landslide instability factors were prepared, including elevation, slope angle, slope aspect, plan curvature, vegetation index, built-up index, stream power, lithology, precipitation intensity, and cumulative precipitation index. Four machine learning algorithms, namely decision tree (DT), support vector machines (SVM), Back Propagation neural network (BPNN), and Long Short Term Memory (LSTM) are implemented, and their final prediction accuracies are compared. The experimental results showed that the prediction accuracies of BPNN, SVM, DT, and LSTM in the test areas are 62.0%, 72.9%, 60.4%, and 81.2%, respectively. LSTM outperformed the other three models due to its capability to learn time series with long temporal dependencies. It indicates that the dynamic change course of geological and geographic parameters is an important indicator in reflecting landslide susceptibility.

## 1. Introduction

The China-Nepal Highway is a vital land route connecting China and Nepal, which is also an important part of the “One Belt and One Road” development strategy. It is located in the Hindu Kush-Himalayan region—one of the most tectonically active regions of the world. Due to the fragile ecological environment and highly-varying hydrothermal conditions, mountain hazards such as landslides and mudslides take place frequently and have caused severe damage to infrastructure. Thus, it is of great importance to perform the mountain hazard assessment in a more accurate and real-time way. Taking landslide related hazards as the research object, a prediction model is established to assess the susceptibility in this paper.

In the past, disaster information extraction and prediction were mainly based on artificial visual interpretation. Apart from being time-consuming and strenuous, the traditional method also has a limitation in that the measurement process lacks of accuracy and depends heavily on experts’ experience. With the development of the computer vision and pattern recognition technologies, it is possible to make the hazard assessment automatic. Synthetic aperture radar (SAR) images have been employed to monitor the surface movement of landslides [1]. Vahidnia et al. [2] applied geographic information systems (GIS) to produce a landslide susceptibility map in which the slope failures that are most likely to happen are displayed. Owing to its high spatial resolution and stereo capability, high-resolution remote sensing images have played an important role in improving the efficiency and accuracy of hazard monitoring [3,4]. The other type of monitoring method is to embed different kinds of sensors related to slope, rainfall, water table level, and other factors into the landslide and sense the dynamic change of signals. Wireless sensor networks are therefore being used to achieve large-scale data collection and transmission [5].

By employing different sensing and monitoring techniques [6,7,8,9], multidimensional and multiscale temporal and spatial data can be collected. Based on the data, a variety kind of models and algorithms have been employed in landslide susceptibility assessment. Statistical regression models are typical methods to directly describe the spatial relationships between landslide occurrence and effecting factors [10,11,12]. Nandi et al. evaluated the multivariate statistical relationship between landslides and various instability factors including slope angle, proximity to stream, soil erodibility, and soil type based on the logistic regression approach [10]. Due to the non-linear condition of hazard prediction, conventional regressive models fail to accurately characterize the causality among variables correctly. Data-driven approaches rely mainly on historical data and do not assume any form of mechanism information, and they have already received much attention in hazard susceptibility assessments, such as support vector machine (SVM), decision tree (DT), neural networks (NN) and so on [13,14,15,16,17,18,19,20,21,22]. Liu et al. developed a hybrid BP neural network to assess the geological hazard risk which adopted genetic algorithm (GA) and particle swarm optimization (PSO) to optimize the network connection weights and thresholds [13]. Marjanović modeled the landslide susceptibility assessment problem as a classification problem, and applied SVM to evaluate which category the region belongs to—stable ground, or dormant and active landslides [4]. As expert experience is helpful to improve prediction accuracy, adaptive neuro-fuzzy inference (ANFI) and Bayesian inference are also widely used in susceptibility assessments [23,24,25,26,27]. Vahidnia employs a fuzzy inference system (FIS) to model expert knowledge, and an artificial neural network (ANN) to assess landslide susceptibility by identifying non-linear behavior and generalizing historical data to the entire region [2]. Chalkias used an expert-based fuzzy weighting (EFW) approach to determine the susceptibility level of different regions by weighted linear combination, in which precipitation, slope, and lithology were considered to be the most important conditioning factors [27].

The formation and occurrence of landslides is a complicated evolution process, which is caused by the interaction of multiple instability factors. However, most of the methods consider only the current value of the instability factors while ignoring the factors’ evolution feature over time. The recurrent neural network (RNN) can use internal memory units to process arbitrary sequences of inputs, thus making RNNs capable of learning temporal sequence. As a special RNN architecture, LSTM inherits RNNs’ good features of sequence learning, and is able to learn the time series with long temporal dependency and automatically determine the optimal result by applying the gate control mechanism. Thus, LSTM has recently attracted wide attention in time series predictions, natural language generation, and so on 28-30]. Ma et al. present a novel LSTM NN to predict travel speed with long time dependencies using microwave detector data. The numerical experiments demonstrate that the LSTM NN outperforms Elman NN, TDNN, and NARX NN in terms of accuracy and stability [28]. Yu developed a transient stability assessment system based on the LSTM network, aiming at balancing the trade-off between assessment accuracy and response time [29]. To our knowledge, Mezaal was the first to use RNN in automatic landslide detection from high-resolution airborne laser scanning data, with an accuracy of more than 80% [30]. In this paper, LSTM is applied to assess the dynamic landslide susceptibility based on multidimensional and multiscale temporal and spatial data. The aim of this research is the assessment of landslide susceptibility based on machine-learning algorithms for the China-Nepal Highway in the Hindu Kush-Himalayan region, taking into consideration the various instability factors and their evolution features.

## 2. Methodology

### 2.1. Study Area

**Figure 1:**
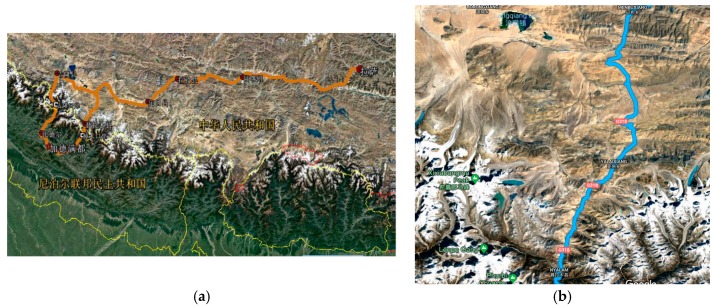
(**a**) map of the China-Nepal Highway; (**b**) Location map of the study area.

The China-Nepal Highway, marked as an orange bold line in Figure 1, is located in the central part of the Hindu Kush Himalayan region (HKH). It runs east to west over 943 km from Lhasa, the capital of Tibet, China, to Kathmandu, capital of the Federal Republic of Nepal. The highway stretches through four large mountains, namely the Tolsan (elevation 4950) and Gatzola Mountain. (elevation 5220), Tonglashan (elevation 5324), Yaxunxiong (elevation 5627), and has an average altitude of more than 4000 m. Due to the fact that the entire area is located in the slope layers and plateau terrain of the Himalayas, the terrain, geology, hydrology, and climate along the highway are extremely complex. Surrounded by high mountains, deep valleys, steep terrain, severe mountain fragmentation, strong new structure movements, frequent earthquakes, and concentrated precipitation (annual rainfall of up to 2500 mm), the highway is heavily affected by natural hazards such as landslides, fragmentation, landslides, and mudslides.

The study area is located in the Nyalam of Shigatse area, where geological disasters occur most frequently. This part stretches 133 km from Mengla in the north to Friendship Bridge bordering Nepal in the south, comprising longitudes 85°57′55″–86°10′7″ and latitudes 27°58′20″–28°48′30″. The topology of the area undulates dramatically, with elevations ranging from 1770 m to 5123 m.

### 2.2. Instability Factors

The first problem to be addressed is the detection of instability factors which cause mountain hazards of different types and degrees. With the development of space techniques and information technologies, a great variety of temporal and spatial data become available, such as geological data, geographic information, high-resolution remote sensing images, hydrological data, and so on. These instability factors can fall into three categories: disaster-causing factors, disaster-pregnant environment factors, and hazard-bearing body factors. A disaster-pregnant environment is characterized by topography, lithology, and the formation of strata, as well as land use. Disaster-causing factors include the precipitation and dynamic change of glacial lakes. The vulnerability degree of hazard-bearing bodies and the dangerous degree of the above two factors together decides the severity of mountain hazard. Since the instability factors are numerous, and most of them have obvious fuzziness and uncertainty, it is difficult to extract key factors that can provide accurate and real-time hazard susceptibility assessment from multi-source data.

**Figure 2:**
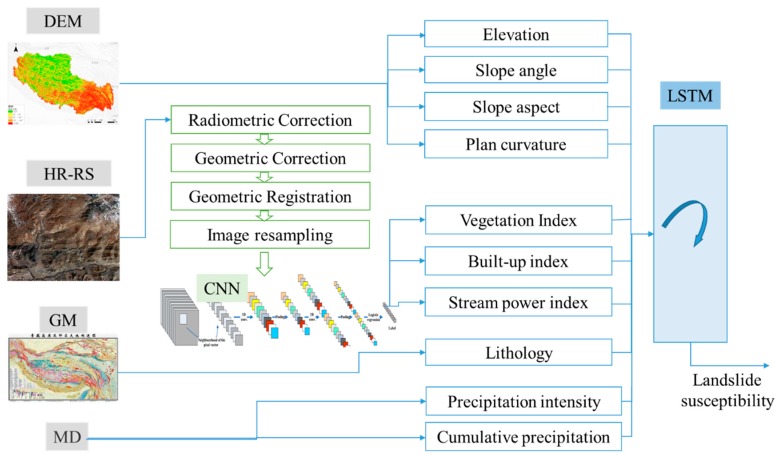
Framework of integrated deep learning-based landslide susceptibility assessment.

Figure 2 illustrates the landslide susceptibility assessment framework based on multi-source data integration and deep learning algorithms. Data sources related to mountain hazards include digital elevation model (DEM), high-resolution remote sensing images (HR-RS), 1: 50,000 geologic maps (GM), and meteorological data (MD). Different features can be extracted from the aforementioned raw data, as follows.

#### 2.2.1. Features Based on DEM

**Figure 3:**
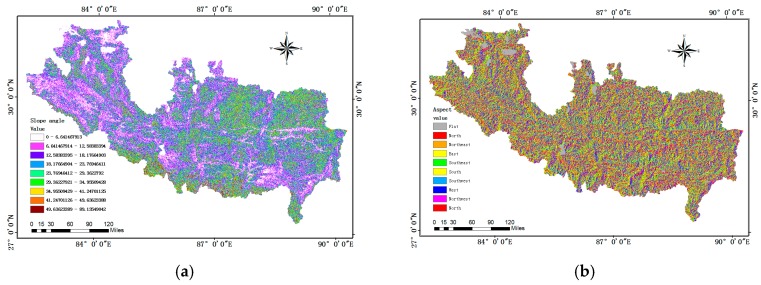

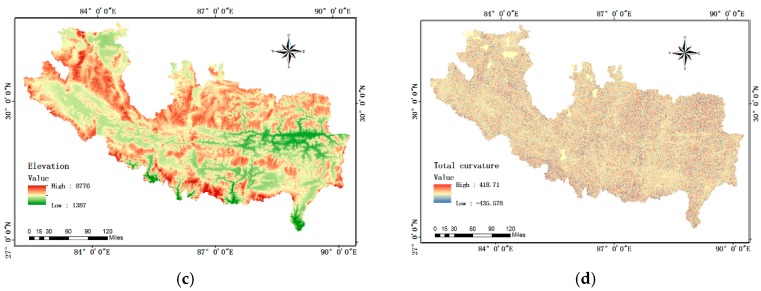
Spatial factors in China-Nepal highway: (**a**) slope angle; (**b**) Slope aspect; (**c**) Elevation; (**d**) Plan curvature.

Slope angle: Slope degree is one of the most frequently-used factors in assessing landslide susceptibility [13,14,15,16,17,18]. It has a great influence on slope stability and is directly related to the different types of mountain hazards (Figure 3a).

Slope aspect: It is defined as the direction of terrain surface, such as north, northeast and so on. Since hillsides orientated differently receive direct solar radiation and rainfall in different amounts, which lead to different slope topography, humidity and plant cover, the slope aspect is also accepted as a conditioning factor (Figure 3b). 

Elevation: Previous records of the China-Nepal Highway hazards indicate that landslides in that area generally occur at a middle elevation (Figure 3c). This is due to the fact that a mountain at high altitudes usually has thin soil cover and a stable rocky structure, while area at low altitudes has gentle slopes, neither of which is susceptible to landslides [13,14]. 

Plan curvature: Curvature is defined as the change rate of slope angle with surface plane. The direction of drainage line is influenced by plan curvature types, and the river erosion is a key factor that affects the slope stability (Figure 3d).

#### 2.2.2. Features Extracted from HR-RS

Remote sensing images are used to extract land cover and utilization information through object-based classification methods. A series of preprocessing work is essential for image classification, including the radiation correction, the geometric correction, the landform correction and the noise reduction. The purpose of radiation correction is to eliminate the difference of spectral reflectivity and spectral radiance between the sensor data and the real images. Geometric correction is the calibration of geometric distortions such as offset, stretching, squeezing, and distortion of the image due to factors such as the rotation or the curvature of the earth, and the temporal and spatial changes of the remote sensing platform. Then different types of land covers are classified, including water, built-up, vegetation, high-way, rock and so on. Cover area of water, vegetation and rock belong to instability factors, while built-up and high-way effect the dangerous degree of landslides. We can obtain four indicators from the classification results: vegetation index, built-up index, road index and stream power index.

#### 2.2.3. Features Based on GM

The development of geological hazards is influenced by strata’s lithology, geological structure and rock-texture. Places with strong structural deformation are easy to form folds and faults, as well as large-scale rock body rupture, which often become the solid source of landslides.

Lithology: The relationship between lithology and solid source is reflected in the weather resistance and anti-erosion ability. Generally, soft layer has low strength and weak resistance to weathering and provides more incompact solid matters. The complex geological structure and the massive loose solid materials intensifies the landslide disaster’s occurring. Geology formations in the study area mainly include limestone, dolomite, sandstone and shale.

#### 2.2.4. Features Based on MD

Water is not only an important component of landslides, but also a triggering condition and transport medium. Rainfall is an important predisposing factor in triggering landslides because it reduces soil suction and increases the pore-water pressure in soils [31,32,33]. Experiments have shown that the landslide occurrence is related both to the intensity and duration of a rainfall event. Thus, two indexes are used to quantify the precipitation characteristics: cumulative precipitation index (CPI) and precipitation intensity index (PII). CPI is calculated with the linear combination of antecedent precipitation in a period, while PII represents the hourly rainfalls which contributes to the landslide-triggering rainfall threshold.

(1) $$P_{a0}=KP_{1}+K^{2}P_{2}+\ldots +K^{n}P_{n}$$

**Figure 4:**
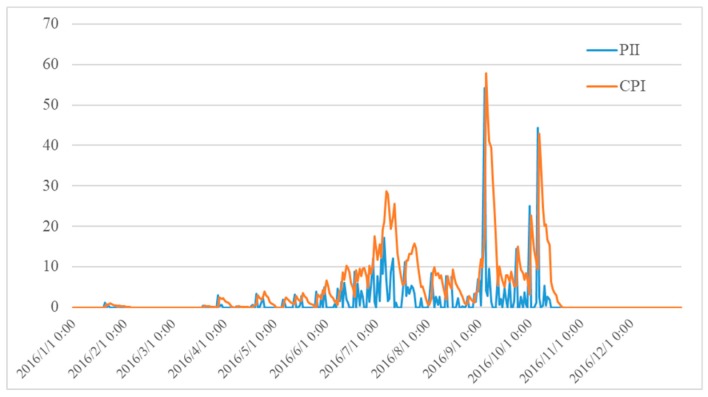
PII and CPI curve of an observation point from 2016/01–2016/12.

$P_{a0}$ was used to define the CPI, where $P_{i}$ is the daily rainfall for the *i*-th day before day 0, *n* is the total number of days considered in the model (*n* = 10 in this work), *K* is the constant decay factor representing the outflow of the regolith (0 < *K* < 1). Figure 4 shows the changing curve of PII and CPI at an observation point during one year from 2016/01–2016/12

### 2.3. LSTM 

**Figure 5:**
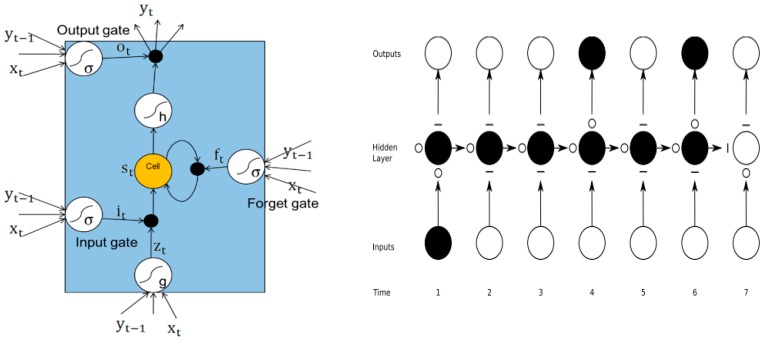
LSTM model structure diagram.

Both SVM and NN belong to the static model, which neglect the dynamic evolution characteristics of mountains and landslide displacement and limit the improvement of prediction accuracy. Unlike the traditional neural network such as BPNN and ANN, RNN adopts recursive connection to construct its internal nodes, so that the state of the previous moment can influence the latter moment, thus realizing the state feedback of the network. However, when the information or time interval between the nodes becomes very long, "It is difficult for RNN to capture long-term time associations, which is called the “vanishing gradient problem”. To solve this problem, LSTM is then proposed by adding a memory block in each unit of hidden layers, which comprises three types of gate functions—input gate, forget gate, and output gate. LSTM uses the memory mechanism to control the transmission of information at different times, which greatly improves the ability of RNN to process long-sequence data. The LSTM model structure diagram is shown in Figure 5.

Input gates:

(2) $$i_{t}=\sigma (W_{xi}x_{t}+W_{hi}h_{t-1}+W_{ci}c_{t-1}+b_{i})$$

Forget gates:

(3) $$f_{t}=\sigma (W_{xf}x_{t}+W_{hf}h_{t-1}+W_{cf}c_{t-1}+b_{f})$$

Cell units:

(4) $$c_{t}=f_{t}c_{t-1}+i_{t}\tanh(W_{xc}x_{t}+W_{hc}h_{t-1}+b_{c})$$

Output gates:

(5) $$\begin{array}{c} o_{t}=\sigma (W_{xo}x_{t}+W_{ho}h_{t-1}+W_{co}c_{t}+b_{o})\\ h_{t}=o_{t}\tanh(c_{t}) \end{array}$$

where $i_{t}$, $f_{t}$, $c_{t}$, $o_{t}$ represents the state vector of the input gate, forget gate, cell unit and output gate at time step t, respectively. $x_{t}$ denotes the input of LSTM network at time t, W is the weight matrix between each layer, h represents the hidden state vector and b is the offset value corresponding to each gate. σ is a sigmoid activation function mapping real numbers to [0,1], while tanh is a hyperbolic tangent function mapping real numbers to [−1,1].

## 3. Results

### 3.1. Four Prediction Models

**Figure 6:**
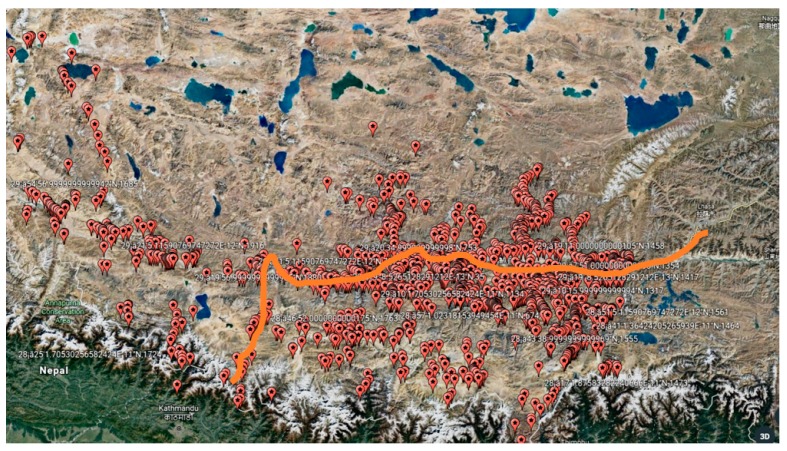
Sample points of test area.

A total of 3800 data points collected from the monitoring site during the period from January 2015 to December 2016 were used in this experiment, which is shown in Figure 6. Data collected between January 2015 and June 2016 were used as a training data set, and the remaining data were used as a test data set. Data preprocessing is performed before the entire data set is split. In order to reduce the influence of the landslide evaluation factor data type, value range, and dimension inconsistency on the prediction model, the original data is normalized to [0,1] closed interval. For each of the attribute values in the evaluation factor, the attribute values are normalized, and the normalization method uniformly uses the range standardization. The sensitivity index was divided into stable, low susceptibility, moderate susceptibility, medium susceptibility and high susceptibility, and very high susceptibility. Thus, the landslide susceptibility assessment is transformed into a classification problem.

Four common classification algorithms are used in the paper to compare with the LSTM model, decision tree (DT), support vector machines (SVM), and Back Propagation neural network (BPNN). The DT and BPNN prediction are performed using Matlab R2013b. LSTM and SVM are implemented in Python using the open source deep learning framework Keras package (which uses TensorFlow as a backend) and the Scikit-learn package, respectively. The parameters of these models are as follows. Table 1 shows the optimal parameters of the four models.

For BPNN, the most widely-used three-layer network consists of an input layer with 10 neurons, one hidden layer with 21 neurons, and one output layer with 1 neuron; it was built as a network structure. The number of hidden layer neurons is determined according to the empirical equation N_h_ = 2N_i_ + 1, where N_h_ represents the number of hidden layer neurons and N_i_ is the number of input layer neurons. Since the initialization weights and thresholds of the BP network have a great influence on the training speed and effect, this paper adopts genetic algorithm to optimize these parameters. 

For SVM, the kernel function is the most important factor determining the model prediction effect. The K-fold Cross Validation (K-CV) method is applied to search the optimal parameters (*K* = 20 in the paper). The original data is divided into *K* groups, of which each subset data is used as a test set and the remaining *K*-1 subset data is used as a training set. By using the K-CV method, the classification accuracies under different combination of c and g are obtained. The combination of c and g with the highest classification of accuracy is selected as the best parameter.

For DT, the purpose of parameter optimization is to prevent the structure of the tree from being too large, resulting in over-fitting problems. Info entropy and gini index are the most commonly-used impurity functions to split the nodes. max_depth and min_samples_leaf act as a constraint to determine the termination of the decision tree construction, thereby controlling the size of the tree.

For LSTM, the length of the input sequence determines the number of the historical data points in the recursive connection. By the grid search method, the input sequence length is set to 8 in this paper.

### 3.2. Experiment Result

As mentioned above, this paper establishes the landslide hazard prediction as a classification problem, and the sample points can be divided into six categories according to different landslide susceptibility levels, i.e., stable, low susceptibility, moderate susceptibility, medium susceptibility and high susceptibility, and very high susceptibility. In the experiment, stable is denoted as label 1, while very high susceptibility is denoted as label 6. Through expert experience and manual judgment, the number of sample points of each susceptibility level is shown in Table 2, where 1612 of 3800 points are in a stable condition, 934 of 3800 points are in a low susceptibility condition, 549 of 3800 points are in a moderate susceptibility condition, 259 of 3800 points are in a medium susceptibility condition, 234 of 3800 points are in a high susceptibility condition, and 212 of 3800 points are in a very high susceptibility condition.

**Figure 7:**
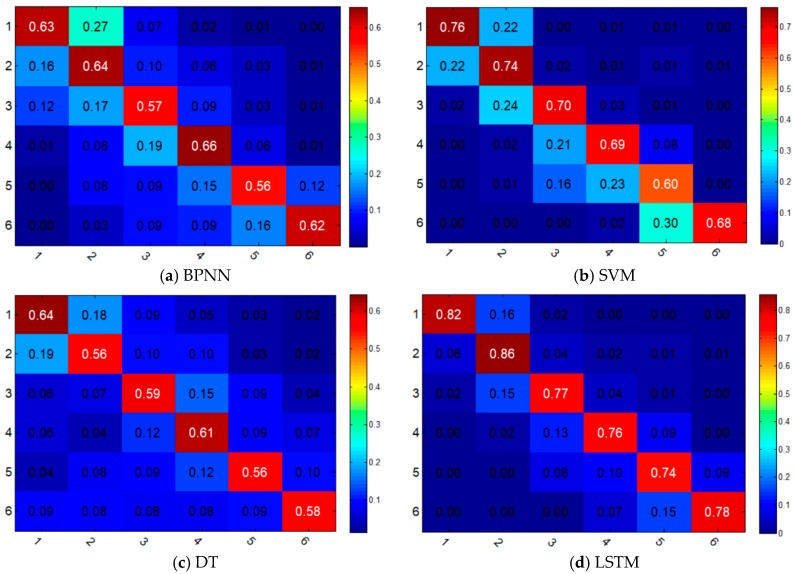
Multi-class confusion matrix of the four models for landslide hazard prediction.

Table 2 and Figure 7 illustrate the prediction results of different classification models. In Table 2, take the first row as an example; it shows that by applying BPNN model, 1015 points are correctly classified into label 1 (stable), which means the accuracy is 62.97%. For the sample points in label 1, BPNN, SVM, DT, LSTM models achieved accuracies of 62.97%, 76.36%, 64.21%, and 82.20%, respectively. Figure 7 shows the confusion matrixes of the four models. It is a visual display tool for evaluating the quality of a classification model, wherein each column of the matrix represents the sample label predicted by the model, while each row of the matrix represents the true label of the sample.

In actual situations, prediction results of the landslide susceptibility level within a certain margin of error are acceptable. For example, if the actual area is in a stable condition and by prediction models it is classified as being in a low susceptibility category, then the prediction results can be considered as acceptable. Thus, in the paper, the prediction error level (PEL) is defined as an indicator to measure the prediction effect of different models.

(6) $${\mathrm{PEL}}_{k}=\frac{\sum_{i=1}^{6}\sum_{J=\max\left(i-k,0\right)}^{J=\min\left(i+k,6\right)}\hat{N_{j}}}{\sum_{i=1}^{6}N_{i}}$$

where ${\mathrm{PEL}}_{k}$ represents the *k*th prediction error level, $\hat{N_{j}}$ is the points number in label *J* of the prediction results, $N_{i}$ is the points number in label *i* of the actual sample.

**Figure 8:**
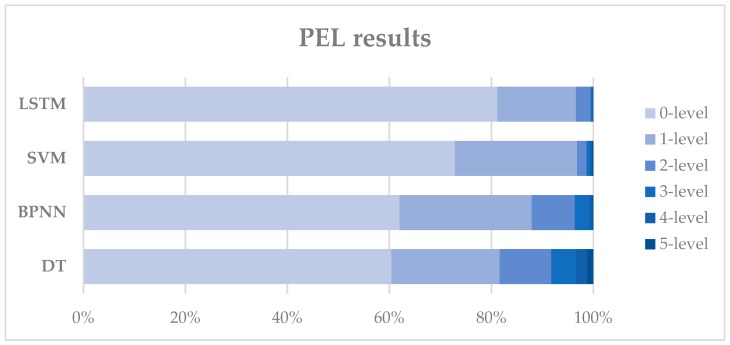
PEL results of the four models.

In Table 3, 0-level represents the prediction accuracy of different labels, while 1-level means the prediction error is only one interval, for example, the actual condition is low susceptibility while the predicted condition is stable or moderate susceptibility. In practice, the prediction results with 0-level or 1-level error are acceptable and can be used to make preventative and control measures. In Figure 8, we can see that almost 90% of the prediction errors of LSTM are 0-level or 1-level. 

## 4. Discussion

The overall prediction accuracies of BPNN, SVM, DT, and LSTM are 62.0%, 72.9%, 60.4% and 81.2%, respectively. As the performance of a data-driven model is greatly affected by the sample size, there are differences in prediction accuracies among different labels. From Table 2, we can also conclude that high susceptibility is the most difficult condition to predict, since it only has an accuracy of 73.5% by LSTM, while the stable condition has an accuracy of 82.2%. In general, LSTM and SVM outperform BPNN and DT in each category in terms of stability of accuracy across different folds of the tested dataset. This is due to the fact that SVM is a structural learning method, which makes it advantageous in solving high dimensional models of small-sample sets. Meanwhile, the historical information from the previous steps contained in the hidden layer of LSTM makes it the most accurate among the four models.

The confusion matrixes in Figure 6 show that there is a certain classification error between label 1 and label 2 for BPNN, SVM, and DT, which means that it is hard for them to distinguish the low susceptibility from the stable condition. Although SVM has a relatively good accuracy, it does not perform well in classifying the neighboring two categories. From this perspective, LSTM is better than SVM since the boundary between the diagonal section and other section in the confusion matrix is obvious. From Figure 8 we can see that the prediction error at level 2 or below of all these four models accounts for more than 90%, which means the four models can predict the landslide susceptibility well within an acceptable error range. The LSTM model has the lowest probability of large prediction errors (3-level or above), while the DT model has the highest probability, which is 0.5% and 8.24%, respectively. We can also conclude that the performance of SVM model is very close to the performance of LSTM, when considering the probability of small prediction errors (1-level or below), which are 96.84% and 96.58%, respectively. 

According to the prediction results, the very high susceptibility dataset has either of the following characteristics: (1) elevation higher than 4000 m, lithology with shales, slope angle from 40° to 55°, and vegetation index lower than 10 (2) elevation from 2000 m to 2800 m, slope angle from 20° to 35°, plan curvature higher than 200 and CPI higher than 30. This result is in accordance with the actual situation. 

## 5. Conclusions

The China-Nepal Highway is an important part of the Belt and Road development strategy. Due to the harsh natural environment along the road, the frequency and intensity of local mountain disasters are increasing, and the casualties and economic losses are increasing accordingly. Therefore, this paper takes the China-Nepal Highway as the research object and conducts risk assessments for mountain disasters. With the development of information and sensing technology in recent years, more and more sensor data and remote sensing data are collected, and a great variety of temporal and spatial data has become available, such as geological data, geographic information, high-resolution remote sensing images, hydrological data, and so on. The influence of various factors on risk has the characteristic of ambiguity, and hierarchies exist between the various degrees of influence. Classical mathematical models are ill-suited to express these complex relationships. At the same time, previous studies only used the static data and characteristics of the study area to characterize the intensity of landslides and debris flow disasters, and these factors have dynamic evolution characteristics. 

To solve this problem, a novel and dynamic model that can remember historical data using so-called “memory blocks” is proposed to solve the problem of the hysteresis effects of triggering factors and landslide susceptibility. The other three classic classification models, BPNN, SVM, and DT, are also applied for comparisons with the LSTM model in landslide susceptibility assessments. The results of this study showed that the SVM model (72.87%) had better accuracy than the BPNN (62.03%) and DT model (60.42%). The LSTM model (81.18%) outperformed SVM in prediction accuracy, and they have the similar performance when considering about the probability of small prediction errors (1-level or below).

## Figures and Tables

**Table 1:** Optimal parameters of different models.

Model	Parameter	Value	Description
BPNN	Number of hidden layer neurons	21	
	Activation function	Sigmoid function	
SVM	c	0.15	Penalty coefficient
	g	0.75	Parameter of RBF
	Kernel function	Radial basis functions	
DT	criterion	Gini	Criterion for feature selection
	max_depth	30	Maximum depth of the tree
	min_samples_leaf	50	Minimum sample number of the leaf node
LSTM	input sequence length	8	
	Loss function	Categorical cross-entropy	

**Table 2:** Classification results of different models.

	Models	Label 1	Label 2	Label 3	Label 4	Label 5	Label 6
Label 1 (1612)	BPNN	1015	434	115	31	13	4
	SVM	1231	351	2	10	10	8
	DT	1035	285	142	84	41	25
	LSTM	1325	251	36	0	0	0
Label 2 (934)	BPNN	154	597	90	56	30	7
	SVM	204	691	16	5	13	5
	DT	179	526	94	94	25	16
	LSTM	56	801	40	19	13	5
Label 3 (549)	BPNN	67	96	312	51	19	4
	SVM	12	133	384	15	5	0
	DT	32	40	322	82	50	23
	LSTM	12	83	423	23	8	0
Label 4 (259)	BPNN	3	20	49	170	15	2
	SVM	0	5	54	179	21	0
	DT	16	11	31	159	23	19
	LSTM	0	4	34	198	23	0
Label 5 (234)	BPNN	0	19	21	35	131	28
	SVM	0	3	38	53	140	0
	DT	10	19	20	29	132	24
	LSTM	0	1	18	23	172	20
Label 6 (212)	BPNN	1	7	19	20	33	132
	SVM	0	0	0	4	64	144
	DT	19	18	17	16	20	122
	LSTM	0	0	0	14	32	166

**Table 3:** PEL results of different models.

	0-Level (Excellent)	1-Level (Good)	2-Level (Moderate)	3-Level (Poor)	4-Level (Bad)	5-Level (Very Bad)
**BPNN**	62.03%	25.92%	8.42%	2.97%	0.53%	0.13%
**SVM**	72.87%	23.97%	1.87%	0.69%	0.40%	0.21%
**DT**	60.42%	21.24%	10.10%	4.85%	2.24%	1.16%
**LSTM**	81.18%	15.40%	2.92%	0.37%	0.13%	0
